# Simvastatin Mitigates Apoptosis and Transforming Growth Factor-Beta Upregulation in Stretch-Induced Endothelial Cells

**DOI:** 10.1155/2019/6026051

**Published:** 2019-12-17

**Authors:** Gang Dong, Xiaoquan Huang, Siyu Jiang, Liyuan Ni, Shiyao Chen

**Affiliations:** Department of Gastroenterology and Hepatology, Zhongshan Hospital, Fudan University, Shanghai, China

## Abstract

Portal hypertension is a common clinical symptom of digestive disorders. With an increase in portal pressure, the portal vein will continue to dilate. We aimed to determine whether continuous stretch induced by portal hypertension may impair the function of endothelial cells (ECs) in the portal vein and aggravate the progress of portal hypertension and explore its mechanism. ECs were cultured on an elastic silicone membrane and subjected to continuous uniaxial stretch. Apoptosis and expression of TGF-*β* in ECs under stretch were measured. We found that sustained stretch induced the apoptosis of ECs in a stretch length-dependent manner. Compared with the control, continuous stretch increased the nicotinamide adenine dinucleotide phosphate oxidase 2 (NOX2) expression and damaged the mitochondria, resulting in an evident increase in reactive oxygen species (ROS) levels; pretreatment with gp91ds-tat or MitoTEMPO decreased the ROS level in the intracellular levels. N-acetyl-cysteine (NAC) treatment before stretch not only reduced ROS levels but also mitigated the apoptosis of ECs; simvastatin had similar effects through targeting NOX2 and mitochondria. During the stretch, the phosphorylation of p38 mitogen-activated protein kinase (P38MAPK), c-Jun N-terminal kinase (JNK), and nuclear factor-kappa B (NF-*κ*B) was obviously increased; pretreatment with P38MAPK or JNK inhibitors decreased the phosphorylation of NF-*κ*B and TGF-*β* expression. Pyrrolidine dithiocarbamate (PDTC) treatment before stretch also reduced TGF-*β* expression. After pretreatment with NAC, the phosphorylation of P38MAPK, JNK, and NF-*κ*B and TGF-*β* expressions in ECs under stretch was suppressed; similar results were observed in simvastatin-treated ECs. This study demonstrated that simvastatin could mitigate EC apoptosis and TGF-*β* upregulation induced by continuous stretch by reducing the level of ROS.

## 1. Introduction

The splenic vein and mesenteric vein are the main branches of the portal vein, which transmits blood from the abdominal organs to the hepatic sinusoids [1]. The normal mean portal vein diameter has been reported about 11 mm among healthy adults [2]; diameters of greater than 13 mm have the tendency to be diagnosed with portal hypertension [3, 4]. Portal hypertension is one of the complications of hepatic fibrosis [5], when portal hypertension develops and fails to receive timely treatment, the portal vein will continue to dilate [6]. The major hemodynamic forces associated with portal hypertension can be divided into shear stress, transmural pressure, and mechanical stretch [7, 8]. Among them, the effect of shear stress and transmural pressure on the pathophysiology of portal hypertension has been widely studied [9, 10]. However, the roles of mechanical stretch in portal hypertension remain unclear.

Previous study has reported that portal vein dilatation induced by embolization contributed to uniaxial continuous stretch in ECs [11]. To simulate the changes in ECs induced by portal vein dilatation under portal hypertension, the stretch apparatus reported in a previous study was applied in our study [11]. It was reported that there was an increase in the production of interleukin 6 (IL-6) in the human umbilical vein endothelial cells exposed to stretch [11]. TGF-*β* plays an important role in the development of liver fibrosis [12]; in this study, we aimed to investigate the effect of stretch on the expression of TGF-*β* in ECs. In a rat model of portal hypertension, the portal vein diameter was increased, the endothelial cell degeneration was detected by electron microscopy [13], and the apoptosis of epithelial cells has been reported to be induced by stretch [14]. In the present study, we speculate that continuous stretch contributes to the increase apoptosis of ECs. As an oral lipid-lowering drug, simvastatin has anti-inflammatory and antioxidative effects [15, 16]. Here, we hypothesize that apoptosis increase and TGF-*β* overproduction in stretch-induced ECs may be alleviated by simvastatin.

In the present study, elastic silicone chambers were used to simulate the effects of continuous stretch on ECs under portal hypertension; apoptosis increase and TGF-*β* overproduction were found in stretch-induced ECs, and ROS was involved in these pathophysiological changes. As a potential candidate for pharmacotherapy of portal hypertension, simvastatin may mitigate apoptosis, and TGF-*β* upregulation of stretch-treated ECs through targeting ROS.

## 2. Materials and Methods

### 2.1. Antibodies and Reagents

Anti-*β*-actin antibody (cat. no. ab8224; Abcam), anti-Akt antibody (cat. no. ab8805; Abcam), anti-p-Akt antibody (cat. no. ab38449; Abcam), anti-human JNK antibody (cat. no. ab179461; Abcam), anti-human P-JNK antibody (cat. no. ab124956; Abcam), anti-p38 mitogen-activated protein kinase (MAPK) antibody (cat. no. ab31828; Abcam), anti-phosphorylated (p-)p38 mitogen-activated protein kinase (P38MAPK) antibody (cat. no. ab4822; Abcam), monoclonal, anti-Bax antibody (cat. no.ab32503; Abcam), anti-Bcl-xL antibody (cat. no. ab32370; Abcam), anti-transforming growth factor beta (TGF-*β*) antibody (cat. no. ab92486; Abcam), anti-nuclear factor-kappa B (NF-*κ*B) antibody (cat. no. ab16502; Abcam), anti-caspase-3 antibody (cat. no. 9662S; Cell Signaling Technology), anti-cleaved caspase-3 antibody (cat. no. 9661S; Cell Signaling Technology), anti-p-NF-*κ*B antibody (cat. no. 3033S; Cell Signaling Technology), anti-p-extracellular signal-regulated kinase (ERK) 1/2 antibody (cat. no. 44-680G; Thermo Fisher Scientific, Inc.), and anti-ERK1/2 antibody (cat. no. 13-6200; Thermo Fisher Scientific, Inc.) were used for Western blot. Ammonium pyrrolidine dithiocarbamate (PDTC), reactive oxygen species (ROS) assay, N-acetyl-cysteine (NAC), One Step TUNEL Apoptosis Assay Kit, and JC-1-Mitochondrial Membrane Potential Assay Kit were purchased from Beyotime Institute of Biotechnology (Jiangsu, China). Simvastatin and MitoTEMPO were obtained from Sigma-Aldrich (St. Louis, MO). gp91ds-tat (sequence: H-Tyr-Gly-Arg-Lys-Lys-Arg-Arg-Gln-Arg-Arg-Arg-Cys-Ser-Thr-Arg-Ile-Arg-Arg-Gln-Leu-NH2) was obtained from AnaSpec (Fremont, USA). Collagen type I was bought from Advanced BioMatrix (San Diego, CA).

### 2.2. Cell Culture and Mechanical Stretch

Endothelial cells, EA.hy926 (ATCC, Manassas, VA, USA), were cultured in the Dulbecco's modified Eagle's medium (DMEM; Gibco; Thermo Fisher Scientific, Inc.) supplemented with 10% fetal bovine serum and 1% penicillin–streptomycin and incubated in humidified atmosphere of 5% CO_2_ at 37°C.

Uniaxial continuous mechanical stretch was used to simulate the dilation of blood vessels. As previously described [11], ECs were seeded on an elastic silicone chamber (this device is 40 × 20 × 10 millimeters and can be stretched to varying degrees) precoated with collagen type I (50 *μ*g/ml); the medium was changed before initiating the stretch. Two ends of the chamber were fixed to a metal frame with a level of distension increase (15% or 20%); the entire chamber with the metal frame was then placed in an incubator.

### 2.3. TUNEL

One Step TUNEL Apoptosis Assay Kit was used to detect the presence of apoptotic cells according to the manufacturer's instructions. Briefly, ECs were fixed in 4% paraformaldehyde before rinsing with PBS and then permeabilized with 0.1% Triton X-100 followed by FITC-labeled TUNEL. Positive cells with green fluorescence were captured under an Olympus fluorescent microscope using 488 nm excitation and 530 nm emission.

### 2.4. Mitochondrial Membrane Potentials Assay

The JC-1 probe was applied to measure the mitochondrial membrane potential (Δ*ψ*m) in ECs according to the manufacturer's instructions. Cells were incubated with JC-1 staining solution (5 *μ*g/mL) for 20 min at 37°C and were rinsed twice with JC-1 staining buffer. The fluorescence intensity of both mitochondrial JC-1 monomers (green fluorescence) and aggregates (red fluorescence) was monitored under an Olympus fluorescent microscope. The Δ*ψ*m was indicated by the ratio of red fluorescence to green fluorescence.

### 2.5. Reverse Transcription-Quantitative Polymerase Chain Reaction (RT-qPCR) Analysis

Total cellular RNA extracted by TRIzol (Invitrogen; Thermo Fisher Scientific, Inc.) was reverse-transcribed into cDNA using a RevertAid First Strand cDNA Synthesis kit (Thermo Fisher Scientific, Inc.) [17]. The primers used were as follows: NADPH oxidase 2 (NOX2): 5′-TTCCAGTGCGTGCTGCTCAAC-3′ (sense) and 5′-TGGTGTGAATCGCAGAGTGAAGTG-3′ (antisense); NOX4: 5′-GTGTCTAAGCAGAGCCTCAGCATC-3′ (sense) and 5′-CGGAGGTAAGCCAAGAGTGTTCG-3′ (antisense); TGF-*β*: 5′-GTACCTGAACCCGTGTTGCT-3′ (sense) and 5′-GTATCGCCAGGAATTGTTGC-3′ (antisense); and Bax: 5′-GATGCGTCCACCAAGAAGCTGAG-3′ (sense) and 5′-CACGGCGGCAATCATCCTCTG-3′ (antisense). The resultant cDNA was amplified using the FastStart Universal Probe Master (Roche Diagnostics) in accordance with the manufacturer's protocol. Target genes were quantified using the 2^−*∆∆*Cq^ method and normalization with the expression of *β*-actin.

### 2.6. Western Blot

Radioimmunoprecipitation assay buffer (RIPA) containing phenylmethylsulfonyl fluoride (PMSF) and phosphatase inhibitor (Beyotime Institute of Biotechnology) were used to lyse cells on ice. A bicinchoninic acid (BCA) protein assay (Pierce; Thermo Fisher Scientific, Inc.) was used to measure protein concentration. Protein samples (20 *μ*g/well) were separated by sodium dodecyl sulphate (SDS) polyacrylamide gel electrophoresis and transferred to a polyvinylidene difluoride (PVDF) membrane (Millipore); approximately 5% low fat milk was used to block the PVDF membrane for 1 h, and primary antibodies were used to incubate it at 4°C overnight. The next day, the PVDF membrane was incubated with secondary antibodies (Beyotime Institute of Biotechnology) for 1 h and then developed by enhanced chemiluminescence (Pierce; Thermo Fisher Scientific, Inc.). The target protein level was normalized to that of *β*-actin, and the phosphorylated protein level was normalized to the corresponding total protein.

### 2.7. ROS Measurement

2′,7′-Dichlorodihydrofluorescein diacetate (DCFH-DA) (Beyotime Institute of Biotechnology) was used to measure ROS. Following stretch, ECs were incubated with 10 *μ*M DCFH-DA for 30 min at 37°C and rinsed with serum-free media. The fluorescence of DCFH-DA-labeled cells was monitored under an Olympus fluorescent microscope.

### 2.8. Statistical Analysis

All data were analyzed using the SPSS 13.0 software (SPSS, Inc.) and expressed as means ± SD. Student's unpaired *t* test was used to compare between two samples. *P* < 0.05 was considered significant.

## 3. Results

### 3.1. Stretch-Induced Apoptosis of ECs

**Figure 1:**
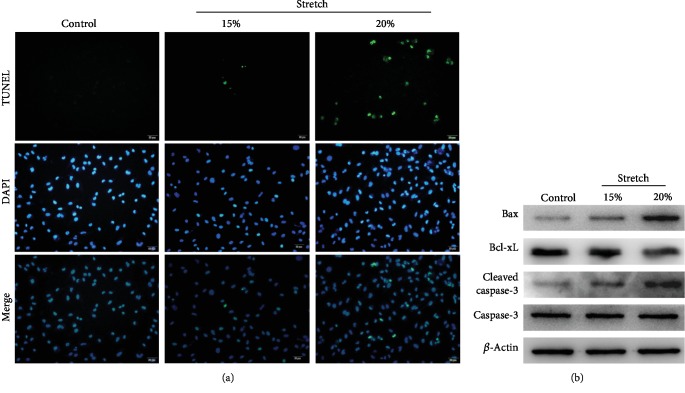
Stretch-induced apoptosis of endothelial cells (ECs). (a) Apoptosis of ECs under nonstretch, 15% stretch, and 20% stretch conditions were measured by TUNEL assay. TUNEL-positive cells were stained into green, while nuclei were counterstained into blue. (b) Proteins associated with proapoptosis and antiapoptosis were detected by Western blot. The target protein was normalized to that of *β*-actin.

ECs were exposed to nonstretch, 15% stretch, and 20% stretch conditions. As shown using TUNEL analysis (Figure 1(a)), ECs when exposed to 15% stretch and 20% stretch had increased apoptotic activity than ECs that were not exposed to stretch. However, the apoptotic activity of ECs exposed to 20% stretch was markedly increased compared with that of ECs exposed to 15% stretch. The results above were further confirmed by Western blot (Figure 1(b)); with an increase in the length of stretch, proteins associated with proapoptosis were induced, and antiapoptotic proteins were correspondingly reduced. These data suggest that sustained stretch can induce apoptosis of ECs in a stretch length-dependent manner; moreover, 20% stretch strikingly increased their apoptotic activity.

### 3.2. Apoptosis of ECs Induced by Stretch via ROS

**Figure 2:**
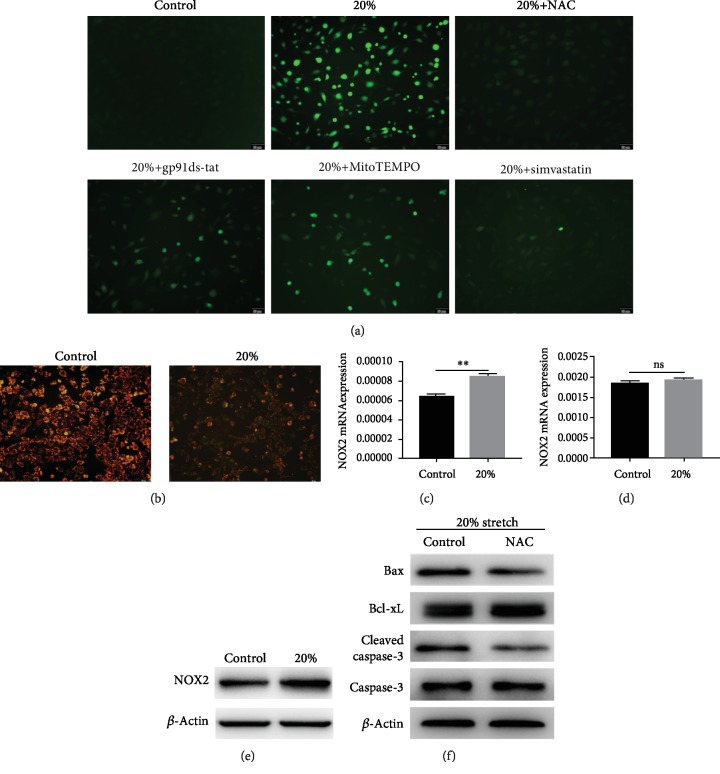
Apoptosis of ECs induced by stretch via reactive oxygen species (ROS). (a) Intracellular ROS in ECs treated by nonstretch, 20% stretch, 20% stretch+N-acetyl-cysteine (5 mM), 20% stretch+gp91ds-tat (5 *μ*M), 20% stretch+MitoTEMPO (10 *μ*M), and 20% stretch+simvastatin (1 *μ*M) and measured by 2′,7′-dichlorodihydrofluorescein diacetate. (b) Mitochondrial membrane potential was detected by a JC-1 probe; the ratio of red to green fluorescence decreased in ECs under 20% stretch. (c–e) The mRNA expression levels of nicotinamide adenine dinucleotide phosphate oxidase 2 (NOX2) and NOX4 were measured using quantitative reverse-transcription polymerase chain reaction (qRT-PCR) analysis in ECs under 20% stretch; the protein level of NOX2 was detected by performing Western blot. (f) Antiapoptotic and proapoptotic proteins of ECs pretreated with or without NAC under 20% stretch were evaluated by performing Western blot. ^∗∗^*P* < 0.01.

Stretch increased the ROS production in the central nervous system injury [18]. Therefore, we investigated whether oxidative stress induced by stretch can trigger the apoptosis of ECs. As shown in Figure 2(a), compared with the control, 20% stretch significantly increased the intracellular levels of ROS, whereas ROS production was markedly decreased by N-acetyl-cysteine (NAC; a ROS scavenger) pretreatment. Mitochondria and nicotinamide adenine dinucleotide phosphate oxidase (NOX) were the major sources of ROS [19]; the mitochondrial membrane potential decreased, and the NOX2 expression of ECs increased under 20% stretch (Figures 2(b), 2(c), and 2(e)). Considering that NOX2 and NOX4 of ECs equally contributed to ROS generation [20], there was no significant difference in the expression of NOX4 before and after exposure to 20% stretch (Figure 2(d)); we chose a selective NOX2 inhibitor (gp91ds-tat) and a mitochondria-targeted antioxidant (MitoTEMPO) to further explore the source of ROS level of ECs exposed to 20% stretch. ROS production was lowered at different levels after pretreatment with gp91ds-tat or MitoTEMPO (Figure 2(a)). To determine whether apoptosis of 20% stretch-treated ECs was induced by ROS, we used NAC to scavenge ROS from different sources; pretreatment with NAC markedly increased the expression of antiapoptotic proteins and decreased the expression of proteins associated with proapoptosis (Figure 2(f)). These data suggest that ROS from NOX2 and the mitochondria increased the apoptosis of ECs under 20% stretch.

### 3.3. Effects of Simvastatin against Endothelial Cell Apoptosis Induced by Stretch

**Figure 3:**
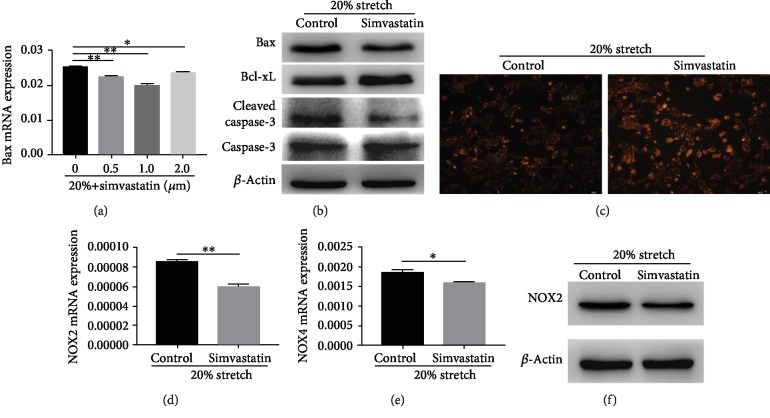
Simvastatin protected ECs from apoptosis induced by stretch. (a) Bax mRNA of ECs under 20% stretch with different concentrations of simvastatin was measured by qRT-PCR analysis. (b) Antiapoptotic and proapoptotic proteins of ECs under 20% stretch with simvastatin or not were detected by Western blot. (c) Reduced mitochondrial membrane potential of ECs under 20% stretch was reversed by simvastatin. (d–f) NOX2 upregulation induced by stretch was inhibited by simvastatin, and even NOX4 expression was reduced. ^∗^*P* < 0.05, ^∗∗^*P* < 0.01.

Simvastatin prevents inflammatory cytokines from injuring endothelial cells [21]. In this study, ECs were pretreated with different concentrations of simvastatin before exposure to 20% stretch (Figure 3(a)). Approximately 1 *μ*M of simvastatin could significantly reduce the Bax (apoptosis-associated gene) expression of ECs induced by stretch, which was further confirmed by Western blot (Figure 3(b)). Next, we investigated whether treatment with simvastatin mitigated EC apoptosis by targeting ROS. As shown in Figure 2(a), treatment with simvastatin markedly reduced the ROS production compared with nontreatment. With regard to the source of ROS, simvastatin pretreatment could not only inhibit NOX2 expression, it could also decrease the expression of NOX4 (Figures 3(d)–3(f)) and restore the mitochondrial membrane potential (Figure 3(c)). These data suggest that simvastatin mitigates ROS-induced apoptosis of ECs under 20% stretch by repression of NOX2 expression and restoration of mitochondrial membrane potential.

### 3.4. Effects of NF-*κ*B in the TGF-*β* Expression of ECs Exposed to Stretch Induced by P38MAPK/JNK

**Figure 4:**
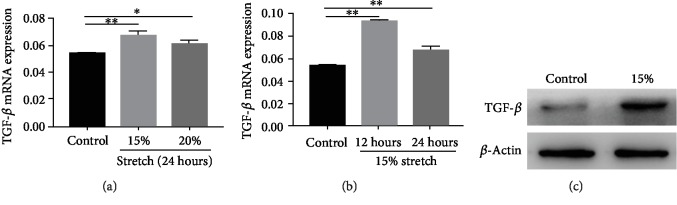
Transforming growth factor beta (TGF-*β*) increased in ECs exposed to stretch. (a, b) TGF-*β* mRNA in ECs under 15% and 20% stretch for 24 h or 15% stretch for 12 h was evaluated by qRT-PCR analysis. (c) TGF-*β* protein in ECs under 15% stretch for 12 h was detected by Western blot. ^∗^*P* < 0.05, ^∗∗^*P* < 0.01.

**Figure 5:**
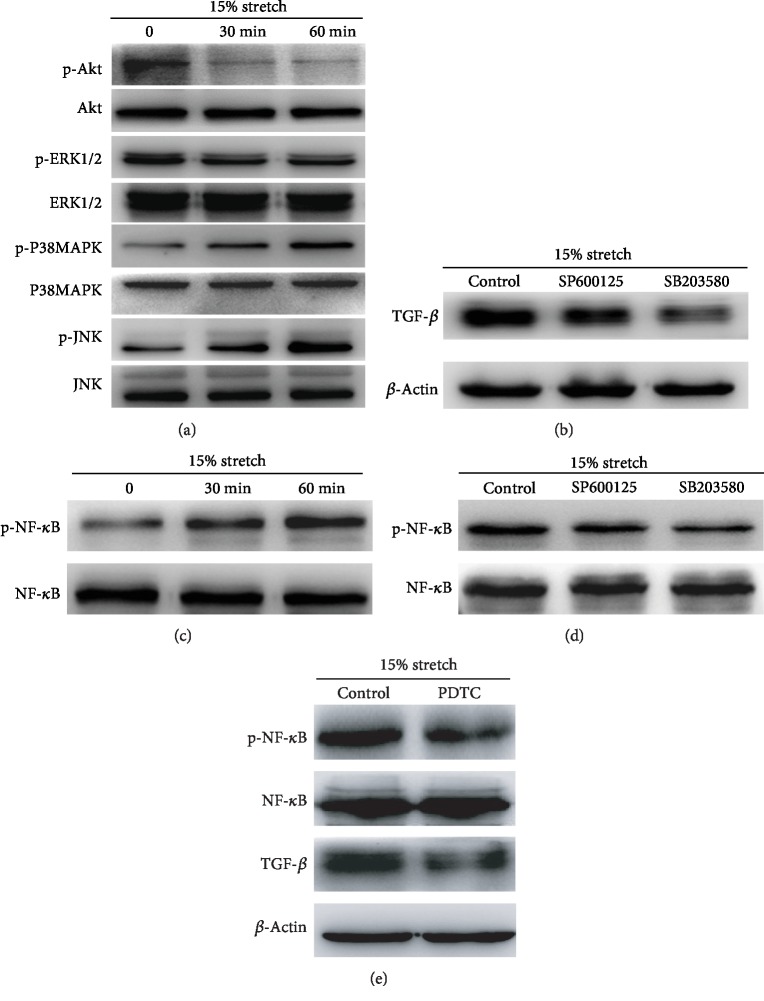
NF-*κ*B mediates TGF-*β* increase in ECs exposed to stretch induced by p38 mitogen-activated protein kinase/N-terminal kinase (P38MAPK/JNK). (a) Stretch activated the P38MAPK and JNK pathways and inactivated the Akt and ERK1/2 pathways in ECs, which was evaluated by Western blot analysis. (b) Stretch-treated ECs were pretreated with SB203580 (a P38MAPK inhibitor) or SP600125 (a JNK inhibitor), the expression of TGF-*β* was significantly decreased. (c) The nuclear factor-kappa B (NF-*κ*B) activity in stretch-treated ECs was evaluated by Western blot analysis. (d) Stretch-treated ECs were pretreated with SB203580 or SP600125, the phosphorylation of NF-*κ*B was significantly inhibited. (e) The phosphorylation of NF-*κ*B and TGF-*β* expression in stretch-treated ECs pretreated with PDTC was remarkably suppressed.

Mechanical stretch has been reported to increase IL-8 secretion [22]; in this study, we explored whether continuous stretch contributes to an increase in TGF-*β* expression. As is shown in Figure 4, both 15% stretch and 20% stretch could upregulate TGF-*β* expression within 24 h compared with the control, especially under 15% stretch within 12 h. Next, the mechanism of TGF-*β* increase induced by stretch was investigated. During stretch, the phosphorylation level of Akt and ERK was decreased; conversely, P38MAPK phosphorylation and JNK phosphorylation were significantly increased (Figure 5(a)). TGF-*β* upregulation induced by stretch was reversed using selective inhibitors of P38MAPK or JNK (Figure 5B). NF-*κ*B was involved in the secretion of IL-8 induced by stretch [23]. As is shown in Figure 5(c), the phosphorylation level of NF-*κ*B increased under stretch, which was inhibited using selective inhibitors of P38MAPK or JNK (Figure 5(d)). The upregulation of TGF-*β* induced by stretch was also suppressed by PDTC (NF-*κ*B inhibitor) (Figure 5(e)). These data show that NF-*κ*B mediates the increase of TGF-*β* expression of ECs under stretch induced by P38MAPK/JNK.

### 3.5. ROS Involved in Stretch-Induced TGF-*β* Increase, which Is Suppressed by Simvastatin

**Figure 6:**
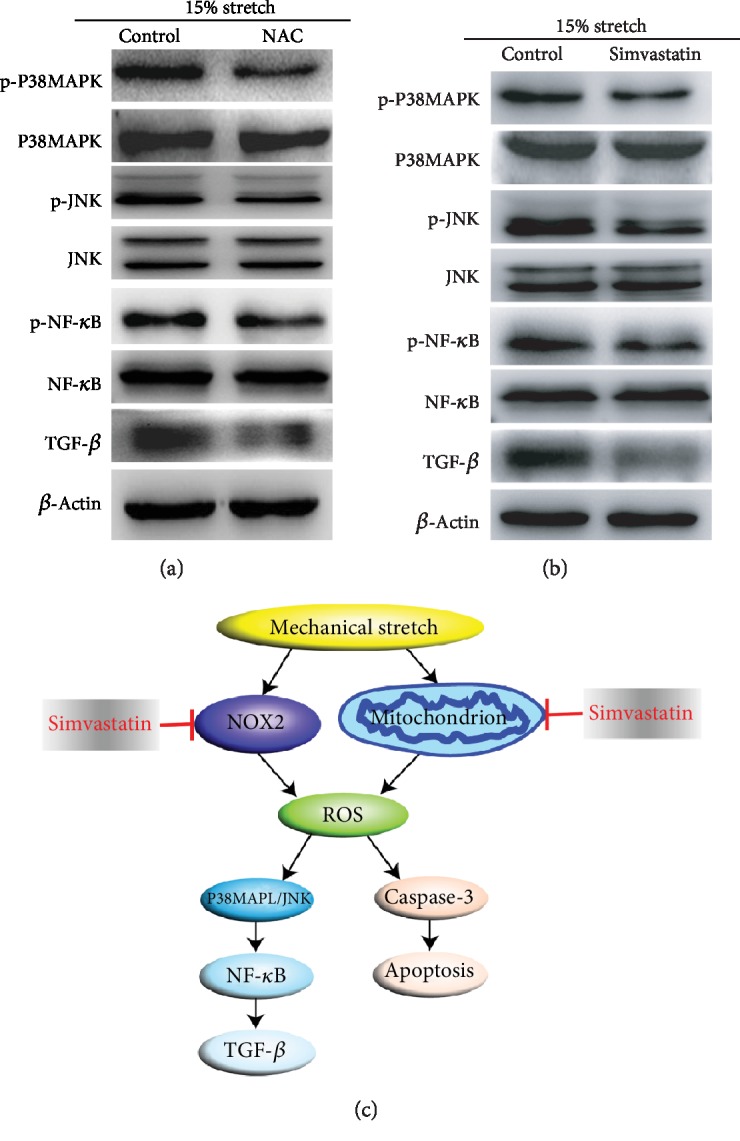
ROS involved in stretch-induced TGF-*β* increase, which was suppressed by simvastatin. (a, b) Stretch-treated ECs were pretreated with N-acetyl-cysteine (NAC) or simvastatin; the phosphorylation of P38MAPK, JNK, and NF-*κ*B and TGF-*β* expressions was significantly suppressed. (c) A graph showed that simvastatin alleviated the apoptosis of ECs and upregulation of TGF-*β* induced by stretch through inhibiting ROS production.

We have confirmed that stretch increased the ROS level; here, we investigated whether ROS was responsible for stretch-induced TGF-*β* increase. As shown in Figure 6(a), NAC decreased the phosphorylation level of P38MAPK, JNK, and NF-*κ*B by scavenging ROS and correspondingly reversed TGF-*β* upregulation. We have verified that simvastatin was effective in decreasing the level of ROS (Figure 2(a)); treatment with NAC downregulated the level of TGF-*β* expression, which was also observed in simvastatin (Figure 6(b)). These data suggest that ROS increased the TGF-*β* expression of ECs under stretch, while simvastatin inhibited the upregulation of TGF-*β* by targeting ROS.

## 4. Discussion

This study mainly explored the mechanism of EC apoptosis and TGF-*β* upregulation induced by stretch; sustained stretch induced apoptosis of ECs in a ROS-dependent manner. Mitochondria and NOX2 were the major sources of ROS; scavenging ROS reduced apoptosis of ECs treated by stretch. NF-*κ*B mediated the increase of TGF-*β* expression of ECs induced by P38MAPK/JNK, which were downstream targets of ROS, and TGF-*β* increase could be suppressed by ROS scavenger. Stretch-induced EC apoptosis and TGF-*β* upregulation could be inhibited by simvastatin pretreatment through targeting ROS.

Portal hypertension is a common clinical syndrome in patients with digestive disorders [24]. With the increase of portal pressure, the portal vein will continue to dilate. To simulate stretch of ECs during portal vein dilation, we used the model of uniaxial continuous stretch in the previous study to investigate the pathophysiological changes of ECs in the portal vein in patients with portal hypertension [11]. Stretch increased the apoptotic activity of epithelial cells [14]; in this study, the apoptotic activity of ECs was increased under stretch (Figure 1). Akt and ERK pathways were closely associated with EC proliferation [25], and their phosphorylation level was significantly inhibited in our study (Figure 5(a)), which further confirmed the results above. ROS plays an important role in the progress of portal hypertension. Decreasing the level of ROS has been reported to improve the endothelial dysfunction and reduce portal pressure in cirrhotic rats with portal hypertension [26]. Inhibition of NOX1/4 with GKT137831 significantly increased portal flow resistance and reduced the portal pressure in rats with partial portal vein ligation [27]. ROS overproduction was reported to be induced by mechanical stretch [28], and this finding is consistent with our data (Figure 2(a)); however, ROS generated during stretch is seldom reported. Mitochondria and NADPH oxidase (NOX) were the major sources of ROS [19]; in the present study, the decrease in mitochondrial membrane potential and increase in NOX2 expression of ECs under stretch were reported (Figures 2(b) and 2(c)); after pretreatment with gp91ds-tat or MitoTEMPO, the level of ROS was decreased (Figure 2(a)), which suggested that ROS might originate from the mitochondria and NOX2 of ECs treated by stretch. As the chief source of free radicals, ROS participates in cell damage and apoptosis [29]; in this study, NAC pretreatment not only reduced the level of ROS but also mitigated the apoptosis of ECs under stretch.

Recent studies have reported that mechanical stretch increased the secretion of inflammatory cytokines such as IL-8 and IL-1*β* [30]. TGF-*β* is closely associated with the occurrence and development of liver fibrosis [12]. In the present study, TGF-*β* upregulation was induced by continuous stretch, especially under 15% stretch at 12 hour (Figure 4). Next, the mechanism of TGF-*β* increase induced by stretch was investigated; P38MAPK-TGF-*β* signaling axis was reported to regulate tumor cell proliferation and Kras-induced senescence [31]. Furthermore, the activation of cardiac *β*3-adrenergic receptors in the cardiomyocytes increases the expression of TGF-*β* via the JNK/c-Jun pathway [32]; in our study, the phosphorylation of P38MAPK and JNK was significantly increased during the stretch. After pretreatment with selective inhibitors of P38MAPK or JNK, TGF-*β* upregulation was reversed (Figures 5(a) and 5(b)), which suggested that P38MAPK/JNK mediated TGF-*β* increase in stretch-induced ECs. NF-*κ*B pathway was activated by mechanical stretch [23], and this finding coincides with our results (Figure 5(c)); however, the level of NF-*κ*B phosphorylation was suppressed after pretreatment with inhibitors of P38MAPK or JNK (Figure 5(d)). As a transcription factor, NF-*κ*B has also been verified to mediate TGF-*β* production regulated by HCV [33]. In addition, inhibition of NF-*κ*B reduced TGF-*β* expression during the resolution of inflammation in vivo [34]. In our study, after pretreatment with PDTC, TGF-*β* increase was obviously inhibited (Figure 5(e)); this finding demonstrates that P38MAPK/JNK-induced NF-*κ*B regulates TGF-*β* expression in stretch-treated ECs. P38MAPK and JNK were activated by ROS; as a ROS scavenger, NAC suppressed the phosphorylation of P38MAPK, JNK, and NF-*κ*B. TGF-*β* expression was also inhibited (Figure 6(a)).

In addition to improving hyperlipidemia syndrome, simvastatin reduced the spinal cord neuronal death through decreasing oxidative stress [35]. Additionally, simvastatin prevented the occurrence of several acute or chronic liver failure-derived complications and increased the survival times of rats with cirrhosis and portal hypertension by increasing hepatic sinusoidal function and reducing portal pressure [36]. In our study, we confirmed that simvastatin could reduce ROS levels induced by continuous stretch by downregulating NOX2 and restoring mitochondrial membrane potential. EC apoptosis and TGF-*β* overexpression were then mitigated.

## 5. Conclusions

In conclusion, as presented in Figure 6(c), the present study demonstrated that simvastatin could mitigate EC apoptosis and TGF-*β* upregulation induced by continuous stretch by reducing the ROS level.

## Data Availability

The data used to support the findings of this study are included within the article.
